# Digital leadership as an environmental determinant of teachers’ cognitive and affective mechanisms in artificial intelligence adoption: an integrated UTAUT2–GETAMEL model

**DOI:** 10.3389/fpsyg.2026.1822713

**Published:** 2026-06-03

**Authors:** Fatma Hümeyra Yücel

**Affiliations:** 1 Department of Educational Administration Ahi Evran University, Kırşehir, Türkiye; 2 Department of Educational Sciences, Ahi Evran University, Kırşehir, Türkiye

**Keywords:** artificial intelligence in education, digital leadership, GETAMEL, self-efficacy, structural equation modeling, teacher behavioral intention, technology-related anxiety, UTAUT2

## Abstract

**Introduction:**

The integration of artificial intelligence (AI) in education depends on teachers’ cognitive, affective, and motivational dispositions toward complex digital tools, yet limited research has examined how school leadership shapes these mechanisms. This study tested an integrated model positioning digital leadership as an environmental antecedent and combining the Unified Theory of Acceptance and Use of Technology 2 (UTAUT2) with the General Extended Technology Acceptance Model for E-Learning (GETAMEL) to explain teachers’ behavioral intention to adopt AI-based educational technologies.

**Methods:**

A cross-sectional survey was administered to 477 teachers working in public and private schools in Türkiye. The model included digital leadership, self-efficacy, anxiety, perceived enjoyment, subjective norm, experience, perceived usefulness, perceived ease of use, attitude, facilitating conditions, habit, price value, and behavioral intention. Measurement and structural models were evaluated using Structural Equation Modeling.

**Results:**

The integrated model demonstrated good model fit and explained 61% of the variance in behavioral intention, outperforming standalone UTAUT2 and GETAMEL models. Digital leadership positively predicted self-efficacy, perceived enjoyment, subjective norm, experience, and behavioral intention, while negatively predicting technology-related anxiety. Self-efficacy, enjoyment, experience, subjective norm, and anxiety influenced perceived usefulness and perceived ease of use. Perceived usefulness, perceived ease of use, and attitude were strong predictors of behavioral intention, while facilitating conditions, habit, and price value also directly contributed to intention.

**Discussion:**

Teachers’ AI adoption is shaped not only by perceived utility but also by emotional regulation, intrinsic motivation, social expectations, and routinization processes. The findings suggest that school leaders should provide hands-on AI training, school-based support structures, low-risk experimentation opportunities, and clear school-level guidance to strengthen teachers’ readiness for AI adoption.

## 1 Introduction

The rapid digital transformation of education has been accelerated by recent advances in Artificial Intelligence (AI), reshaping instructional design, assessment practices, and school management worldwide (Fullan et al., 2024; Suryanarayana et al., 2024). AI-based educational technologies increasingly support personalized learning, automated assessment, adaptive feedback, and data-driven decision-making, thereby expanding expectations placed on teachers and school leaders. However, beyond technological innovation, AI integration introduces substantial cognitive, affective, and motivational demands on teachers, requiring them to reinterpret professional roles, develop new competencies, and regulate emotional responses to complex systems.

In the present study, AI-based educational technologies refer to a broad range of tools used for instructional and assessment purposes, including generative AI applications (e.g., ChatGPT), learning analytics systems, automated assessment and grading tools, and adaptive or intelligent tutoring systems. These technologies differ in both pedagogical function and implementation complexity: generative AI may offer a relatively accessible entry point for experimentation, whereas learning analytics and adaptive AI often require greater data literacy, pedagogical redesign, and institutional support. AI-based educational tools also include intelligent tutoring, learning-to-code supports, predictive analytics, and AI-driven content-generation or game-based environments, which differ in complexity and pedagogical affordances (Ateş, 2025; Hartley et al., 2024; Jamarani et al., 2024; Perez-Liebana et al., 2019).

Emerging research indicates that AI technologies evoke stronger psychological reactions than conventional digital tools, including uncertainty, technology-related anxiety, and concerns about professional adequacy (Konca et al., 2025). Teachers’ willingness to adopt AI therefore depends not only on perceived utility but also on self-efficacy beliefs, emotional regulation, social expectations, and habitual engagement patterns. Understanding AI adoption thus requires a framework that integrates cognitive evaluations, affective responses, and environmental influences. Recent studies similarly show that teachers’ AI adoption is shaped by leadership, educational support, teaching concerns, prior experience, and discipline-specific readiness across different educational contexts (Acosta-Enriquez et al., 2025; Aldraiweesh and Alturki, 2025; Du and Gao, 2022; Gamlem et al., 2025; Xue et al., 2025).

In school contexts, leadership represents a central environmental factor shaping teachers’ psychological adaptation to technological change. Digital leadership refers to school principals’ capacity to articulate a clear digital vision, provide professional development, ensure technological infrastructure, and encourage pedagogical innovation (Lin, 2024; Berkovich and Hassan, 2024). Such leadership practices influence teachers’ beliefs about the value, feasibility, and legitimacy of AI use in teaching and assessment. Without strong digital leadership, even advanced AI tools risk superficial adoption or misalignment with instructional goals (Hossain et al., 2025). Moreover, school leaders play a pivotal role in shaping innovation culture, access to resources, and professional learning opportunities, all of which shape teachers’ cognitive, affective, and motivational dispositions toward AI adoption (Işik, 2025; Schmitz et al., 2023).

At the individual level, technology acceptance models offer important theoretical foundations for understanding teachers’ behavioral intentions. The Unified Theory of Acceptance and Use of Technology 2 (UTAUT2) explains adoption through cognitive and contextual determinants such as perceived usefulness, perceived ease of use, social influence, facilitating conditions, hedonic motivation, price value, and habit (Venkatesh et al., 2012). Complementing this perspective, the General Extended Technology Acceptance Model for E-Learning (GETAMEL) incorporates psychological and experiential determinants, including self-efficacy, anxiety, attitudes, and prior experience (Abdullah and Ward, 2016). These constructs are particularly relevant for AI technologies, which often require advanced competence and evoke heightened emotional responses (Konca et al., 2025).

Despite their explanatory value, UTAUT2 and GETAMEL are rarely integrated in studies examining AI-based educational technologies, and limited attention has been given to how digital leadership shapes the cognitive and affective mechanisms embedded within these models (Ateş and Gündüzalp, 2025; Konca et al., 2025). Existing research frequently conceptualizes leadership as contextual support rather than examining how principals’ digital leadership directly influences teachers’ internal belief systems and emotional processes, such as perceived usefulness, perceived ease of use, self-efficacy, or technology-related anxiety (Gkanatsiou et al., 2025). Given that school leaders strongly influence access to resources, professional learning, and innovation culture (Schmitz et al., 2023), overlooking leadership as an active environmental determinant represents a critical theoretical gap in AI adoption research.

Three gaps motivate the present study. First, prior studies on teachers’ AI adoption have typically applied UTAUT2, TAM, or related models separately rather than integrating cognitive–affective and contextual determinants within a unified framework. Second, digital leadership has often been treated as contextual support, but rarely modeled as an environmental antecedent influencing specific psychological mechanisms such as self-efficacy, anxiety, perceived enjoyment, and subjective norm. Third, although teachers’ readiness is frequently discussed in AI adoption research, it is seldom clarified whether readiness refers to a single measurable construct or to a constellation of cognitive–affective dispositions. The present study addresses these gaps by integrating Digital Leadership, UTAUT2, and GETAMEL in a single SEM framework.

To address this limitation, the present study examines how school principals’ digital leadership influences teachers’ intentions to adopt AI-based educational technologies for instructional and assessment purposes. By integrating digital leadership with UTAUT2 and GETAMEL into a unified framework, the study adopts a multilevel psychological perspective that captures organizational, cognitive, affective, and contextual determinants of AI adoption. In doing so, it conceptualizes digital leadership not merely as administrative strategy, but as an environmental influence shaping teachers’ cognitive appraisals, emotional regulation, and motivation-related dispositions.

Accordingly, the study addresses the following research questions:

*RQ1*: How does digital leadership influence teachers’ psychological perceptions related to AI adoption, including perceived usefulness, perceived ease of use, self-efficacy, anxiety, subjective norm, and facilitating conditions?

*RQ2*: How do UTAUT2 and GETAMEL constructs collectively explain teachers’ behavioral intentions to adopt AI-based educational technologies in instructional and assessment practices?

*RQ3*: To what extent does an integrated digital leadership–UTAUT2–GETAMEL model enhance explanatory power for teachers’ behavioral intention toward AI adoption?

This study makes three contributions. First, it integrates Digital Leadership with UTAUT2 and GETAMEL in a single framework, rather than examining these perspectives in isolation. Second, it specifies the psychological pathway through which leadership operates by linking leadership to self-efficacy, anxiety, perceived enjoyment, subjective norm, and subsequent usefulness/ease-of-use appraisals. Third, it interprets teachers’ psychological readiness for AI adoption not as a standalone latent variable, but as a cognitive–affective configuration reflected in these interrelated mechanisms. Practically, this framework also provides a more specific basis for leadership actions, which are later discussed in terms of training, school-based support, low-risk experimentation, and incentive structures.

## 2 Literature review and hypothesis development

### 2.1 Digital leadership and AI-based educational technologies

Ongoing digital transformation has positioned school principals as key actors in shaping how emerging technologies, particularly AI, are integrated into teaching and assessment (Bixler and Ceballos, 2025). Digital leadership involves articulating a strategic digital vision, allocating resources, supporting professional learning, and fostering a culture that encourages innovation and experimentation (Karakose et al., 2024; Xu, 2025). Related research on digital academic leadership and technology-enabled organizational change also emphasizes the role of leadership practices and socio-emotional enablers in technology adoption (Jing et al., 2025; van Dun and Kumar, 2023) Such leadership practices strongly influence teachers’ willingness to engage with advanced technologies by shaping perceptions of feasibility, value, and institutional support (Berkovich and Hassan, 2024). From a Social Cognitive Theory perspective, digital leadership can be conceptualized as an environmental determinant that influences teachers’ cognitive appraisals and emotional regulation processes during AI adoption. By structuring expectations, modeling technology use, and providing mastery-supportive conditions, leaders shape how teachers interpret technological demands and assess their own capability to respond effectively.

As AI applications increasingly support adaptive instruction, automated assessment, and data-driven decision-making (Er-Rafyg et al., 2024; Gambo et al., 2025), successful implementation depends heavily on teachers’ readiness, confidence, and competence (Choi et al., 2025). However, teachers often face barriers including limited experience, insufficient training, ethical concerns, and technology-related anxiety (Li et al., 2022; Liu J. et al., 2025; Liu Y. et al., 2025). These barriers are not solely technical but psychological, reflecting uncertainty appraisals, diminished self-efficacy beliefs, and affective responses to complexity. In this context, principals’ digital leadership plays a critical role in reducing adoption barriers by providing resources, professional development, and collaborative learning opportunities that enhance teachers’ confidence and engagement with AI (Dasruth et al., 2024; Chen and Delaney, 2025). Such practices may attenuate threat perceptions, strengthen mastery beliefs, and foster intrinsic motivation toward experimentation with AI tools. Consistent evidence shows that strong digital leadership promotes technology acceptance and sustainable implementation, whereas weak leadership results in fragmented or superficial adoption (Kovacevic et al., 2025).

### 2.2 Conceptual framework and hypotheses

Building on this foundation, the present study proposes an integrated framework combining digital leadership, UTAUT2, and GETAMEL to explain teachers’ behavioral intentions to adopt AI-based educational technologies. Within this framework, digital leadership is conceptualized at the environmental level as a contextual antecedent influencing teachers’ cognitive and affective belief formation. Rather than functioning merely as administrative support, digital leadership is theorized to shape how teachers interpret technological demands, evaluate their own capabilities, and regulate emotional responses toward AI integration.

From this perspective, digital leadership functions as a primary environmental determinant that shapes teachers’ cognitive–affective dispositions toward AI adoption by reducing uncertainty and strengthening perceptions of support, usefulness, and ease of use (Okunlola and Naicker, 2025; Sagbas et al., 2023). Specifically, leadership practices are expected to directly influence behavioral intention while also exerting indirect effects through key psychological mechanisms, including self-efficacy (capability beliefs), anxiety (threat appraisal), subjective norm (perceived social expectations), and perceived enjoyment (intrinsic motivation). In the present study, psychological readiness is not modeled as a single standalone construct. Rather, it is conceptualized as an interpretive cognitive–affective configuration reflected in teachers’ self-efficacy, anxiety, perceived enjoyment, subjective norm, and related usefulness/ease-of-use appraisals toward AI adoption.

At the cognitive–affective level, GETAMEL contributes psychological and experiential determinants—such as self-efficacy, anxiety, enjoyment, subjective norm, and experience—that shape teachers’ internal evaluations of AI tools (Abdullah and Ward, 2016; Konca et al., 2025). These constructs represent belief-based and emotional processes through which teachers form judgments about AI’s usefulness and usability. UTAUT2 complements this perspective by incorporating contextual and motivational determinants, including facilitating conditions, price value, and habit, which influence sustained and routinized technology use (Basarmak and Ates, 2026; Venkatesh et al., 2012; Zaim et al., 2024). Within the integrated framework, these factors are conceptualized as shaping the consolidation of intention into stable behavioral tendencies.

Across the integrated model, perceived usefulness, perceived ease of use, and attitude operate as central mediating mechanisms linking environmental influences, cognitive–affective processes, and contextual conditions to teachers’ behavioral intention. In particular, digital leadership is expected to influence behavioral intention both directly and indirectly through sequential pathways in which environmental support shapes self-efficacy, anxiety, social norms, and enjoyment, which in turn influence perceived ease of use and perceived usefulness, ultimately forming attitudes and behavioral intention. Teachers are more likely to adopt AI technologies when they cognitively appraise them as useful and manageable and when affective responses such as confidence and enjoyment outweigh anxiety, within a supportive leadership context (Ateş and Gündüzalp, 2025).

#### 2.2.1 Digital leadership and hypothesis development

School principals’ digital leadership is a key environmental determinant of teachers’ cognitive–affective readiness for adopting AI-based educational technologies that require both technical competence and pedagogical adaptation (Dasruth et al., 2024). Digital leadership encompasses articulating a clear digital vision, embedding innovation within school strategy, providing professional development, ensuring technological infrastructure, and encouraging experimentation (Vermeulen et al., 2015). In this study, digital leadership actions refer specifically to articulating a digital vision, modeling AI-related practices, allocating resources, organizing professional learning, creating psychologically safe opportunities for experimentation, and establishing supportive expectations for school-wide AI use. Through these practices, principals shape the psychological climate in which teachers interpret technological demands, form capability beliefs, and regulate emotional responses toward AI integration (Alkan et al., 2025).

Digital leadership is expected to influence adoption intentions through several cognitive and affective mechanisms. First, strong leadership communicates clear institutional expectations and reinforces collective norms, thereby strengthening teachers’ subjective norm, defined as perceived social pressure to engage in AI use (Harsanti et al., 2025). When principals explicitly endorse AI integration and model its value, teachers are more likely to perceive AI adoption as socially legitimate and professionally expected.

Second, by providing access to resources, time, and hands-on learning opportunities, digital leadership enhances teachers’ experience with AI and related technologies. Repeated exposure and guided practice contribute to familiarity and reduce ambiguity, facilitating more favorable cognitive appraisals of AI tools. Experience, in turn, supports belief consolidation and increases confidence in navigating technological demands.

Third, supportive leadership practices may enhance perceived enjoyment, reflecting intrinsic motivation toward technology use. When leaders create psychologically safe environments that encourage experimentation without fear of evaluation, teachers are more likely to engage in curiosity-driven exploration of AI tools. Such intrinsically motivating conditions can strengthen positive affective responses, which are known to facilitate technology acceptance.

In addition, digital leadership plays a central role in shaping teachers’ emotional regulation processes. AI technologies often trigger uncertainty and complexity-related stress; thus, leadership behaviors that provide guidance, structured professional learning, and visible modeling of competence can reduce technology-related anxiety, conceptualized as a threat appraisal response to perceived difficulty (Farmanesh et al., 2025). Simultaneously, mastery-oriented leadership practices can strengthen self-efficacy, defined as teachers’ beliefs in their capability to successfully use AI tools (Omar and Ismail, 2021). By reinforcing mastery expectations and providing vicarious learning opportunities, principals enhance teachers’ confidence in handling technological challenges.

Taken together, digital leadership is theorized to influence behavioral intention both directly and indirectly through its effects on perceived social pressure (subjective norm), experiential familiarity (experience), intrinsic motivation (enjoyment), threat appraisal (anxiety), and mastery expectations (self-efficacy). Accordingly, the following hypotheses are proposed:

*H1*: Digital leadership positively influences subjective norm.

*H2*: Digital leadership positively influences experience.

*H3*: Digital leadership positively influences perceived enjoyment.

*H4*: Digital leadership negatively influences anxiety.

*H5*: Digital leadership positively influences self-efficacy.

*H6*: Digital leadership positively influences teachers’ behavioral intention to adopt AI technologies.

#### 2.2.2 General extended technology acceptance model for educational technologies

The General Extended Technology Acceptance Model for E-Learning (GETAMEL; Abdullah and Ward, 2016) is incorporated to explain the psychological, affective, and experiential mechanisms underlying teachers’ adoption of AI-based educational technologies. GETAMEL extends earlier acceptance models by integrating constructs that reflect the complex emotional and competence-related demands of technology use in educational contexts, making it particularly suitable for AI adoption research (Konca et al., 2025; Özdemir and Ateş, 2025; Zhang and Yang, 2025). Within the proposed framework, GETAMEL functions as an internal mechanism through which digital leadership and contextual supports shape teachers’ behavioral intentions.

From a cognitive appraisal perspective, GETAMEL constructs capture how teachers interpret, evaluate, and emotionally respond to AI technologies. Adoption decisions are shaped not only by instrumental assessments of utility but also by teachers’ appraisals of competence, social expectations, and perceived threat. In this sense, AI adoption involves ongoing emotional regulation processes in which confidence, enjoyment, and anxiety influence how technological demands are perceived and acted upon.

Experience and subjective norm capture teachers’ familiarity with AI tools and the perceived social expectations surrounding their use. More broadly, technology designs and implementation practices that are both enjoyable and appropriately challenging may strengthen intrinsic motivation and engagement (David and Weinstein, 2024). Prior experience with digital or AI-enhanced technologies strengthens teachers’ confidence and increases perceived usefulness and ease of use, while supportive collegial and leadership environments reinforce positive evaluations of AI (Celik et al., 2025; Dringó-Horváth et al., 2025). Perceived enjoyment represents intrinsic motivation and has been shown to enhance both usefulness and ease-of-use perceptions (Wang et al., 2025). In contrast, anxiety related to technological complexity or professional uncertainty can undermine acceptance by intensifying threat appraisals, whereas self-efficacy enhances openness to AI by strengthening confidence in one’s capability to use these tools effectively (Frenkenberg and Hochman, 2025; Herzallah and Makaldy, 2025). Because AI anxiety may involve multiple psychological factors, including uncertainty, perceived dependence, and fear of negative consequences, it is especially important to model anxiety as a distinct affective determinant in AI adoption (Kim et al., 2025). Attitude toward AI, formed through these cognitive and affective evaluations, directly predicts teachers’ behavioral intention to integrate AI into instructional and assessment practices (An et al., 2023).

Based on GETAMEL, the following hypotheses are proposed:

*H7*: Subjective norm positively influences perceived usefulness.

*H8*: Subjective norm positively influences perceived ease of use.

*H9*: Experience positively influences perceived usefulness.

*H10*: Experience positively influences perceived ease of use.

*H11*: Perceived enjoyment positively influences perceived usefulness.

*H12*: Perceived enjoyment positively influences perceived ease of use.

*H13*: Anxiety negatively influences perceived usefulness.

*H14*: Anxiety negatively influences perceived ease of use.

*H15*: Self-efficacy positively influences perceived usefulness.

*H16*: Self-efficacy positively influences perceived ease of use.

*H17*: Perceived ease of use positively influences perceived usefulness.

*H18*: Perceived ease of use positively influences attitude.

*H19*: Perceived usefulness positively influences attitude.

*H20*: Perceived usefulness positively influences behavioral intention.

*H21*: Attitude positively influences behavioral intention.

#### 2.2.3 Unified theory of acceptance and use of technology 2

In addition to GETAMEL, this study incorporates the Unified Theory of Acceptance and Use of Technology 2 (UTAUT2) to capture contextual and motivational determinants of teachers’ adoption of AI-based educational technologies. UTAUT2 extends the original UTAUT by including hedonic, economic, and habitual dimensions of technology use, offering a comprehensive framework for understanding how teachers cognitively evaluate AI tools and how behavioral tendencies become consolidated over time (Venkatesh et al., 2012; Hu et al., 2025). Integrating UTAUT2 with digital leadership and GETAMEL strengthens the explanatory power of the model by simultaneously addressing environmental influences, cognitive–affective processes, and behavioral consolidation mechanisms.

Within UTAUT2, price value reflects teachers’ cognitive cost–benefit appraisal of AI adoption, representing the extent to which perceived instructional and assessment benefits outweigh the anticipated financial, temporal, and cognitive effort required (Elyakim, 2025). In educational contexts, this appraisal process involves judgments about workload, mental effort, and pedagogical return on investment rather than purely monetary considerations. Although many teachers in public schools do not directly purchase AI technologies themselves, price value in the present study should not be interpreted as limited to personal monetary expenditure. Rather, it reflects teachers’ perceived net value regarding whether the instructional and assessment benefits of AI justify the time demands, cognitive effort, workflow adjustments, and school-level resource implications associated with adoption. In line with the operationalization adopted in this study, price value therefore captures a broader evaluation of whether AI use is worthwhile relative to teachers’ professional demands and school resources. In early-stage AI integration, such appraisals may also include indirect costs such as learning time, output verification, and lesson redesign. Facilitating conditions capture teachers’ perceptions of environmental support, including the availability of technical infrastructure, guidance, and institutional assistance (Chou et al., 2024). Psychologically, perceived support reduces uncertainty and lowers perceived barriers, thereby strengthening the translation of intention into sustained engagement. Habit represents the degree of behavioral automaticity and routinization associated with AI use (Biswas and Murray, 2024). When technology use becomes habitual, it requires less conscious deliberation and cognitive effort, reflecting the internalization of AI practices into teachers’ professional routines. Such automaticity reduces resistance and stabilizes continued use over time.

Accordingly, the following hypotheses are proposed:

*H22*: Price value positively affects teachers’ behavioral intention to adopt AI-based educational technologies.

*H23*: Habit positively affects teachers’ behavioral intention to adopt AI-based educational technologies.

*H24*: Facilitating conditions positively affect teachers’ behavioral intention to adopt AI-based educational technologies.

**Figure 1:**
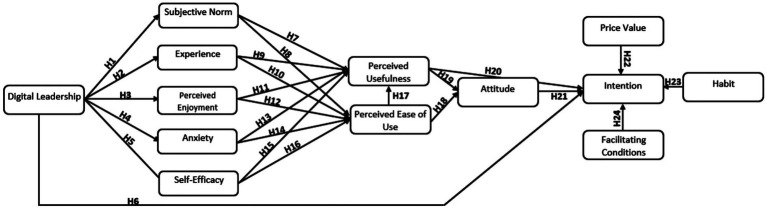
Proposed integrated digital leadership–UTAUT2–GETAMEL model for AI adoption.

Given the limited research integrating digital leadership, GETAMEL, and UTAUT2 in AI-focused educational contexts, this study adopts a comprehensive, theory-driven approach to hypothesis development. Figure 1 presents the proposed integrated model, illustrating how digital leadership functions as an environmental antecedent, GETAMEL captures cognitive and affective appraisal mechanisms, and UTAUT2 explains contextual support and behavioral routinization processes that together shape teachers’ intentions to integrate AI into instructional and assessment practices.

## 3 Method

### 3.1 Data collection and sample

The study was conducted in accordance with the Declaration of Helsinki, and all participants provided informed consent prior to participation. Data were collected through a cross-sectional online survey administered to teachers working in public and private primary, lower secondary, and upper secondary schools in Türkiye during the 2025 academic year. A non-probability convenience sampling strategy was employed. This approach was adopted because the study aimed to test a theory-driven SEM model rather than to generate population estimates, and there was no practical sampling frame that would allow systematic access to teachers with varying levels of AI-related school experience within the limited data-collection period. Distribution through school principals enabled access to teachers across different school levels and institutional contexts and supported the recruitment of a sufficiently large and heterogeneous sample for SEM. However, this approach does not ensure statistical representativeness and may overrepresent teachers or schools that were more willing to engage with digital innovation. Ethical approval was obtained from the relevant institutional review board, followed by permission from educational authorities. School principals were contacted to explain the study’s purpose and voluntary nature, and those who agreed facilitated data collection by distributing the survey link through internal communication channels.

The survey included an informed consent form, demographic questions, and validated scale items measuring Digital Leadership, UTAUT2 and GETAMEL constructs, and behavioral intention toward AI-based educational technologies. Prior to full deployment, the instrument was pilot-tested with 255 teachers to assess clarity and internal consistency. Minor wording revisions were made based on feedback, and pilot data were excluded from the final analysis. The main data collection lasted 4 weeks, with periodic reminders sent to improve response rates. Participation was voluntary and anonymous.

A total of 512 teachers responded to the survey. After data screening, 35 responses were excluded due to excessive missing data or eligibility issues. Cases with more than 10% missing responses were removed from the dataset. Data were further screened for univariate and multivariate normality using skewness and kurtosis statistics, and potential multivariate outliers were examined using Mahalanobis distance values. No extreme outliers were retained in the final dataset. The resulting sample consisted of 477 teachers, which satisfied recommended sample size criteria for structural equation modeling.

The sample was demographically diverse: 58.5% female and 41.5% male, with most participants aged between 31 and 50. The majority held a bachelor’s degree (71%), while 29% had postgraduate qualifications. Teaching experience was substantial, with over half of the participants reporting more than 10 years of professional experience across a broad range of subject areas.

Regarding technology use, most teachers (83%) reported regular use of general digital tools such as interactive whiteboards, learning management systems, and digital assessment platforms. In contrast, direct experience with AI-based educational technologies was limited: only 18% had used at least one AI tool in teaching or assessment, while 82% reported little or no prior AI experience. This profile highlights the relevance of the sample for examining early-stage AI adoption intentions in school settings.

### 3.2 Data collection tools

Data were collected using a structured questionnaire aligned with the study’s conceptual framework. The instrument measured teachers’ perceptions of school principals’ digital leadership, technology acceptance beliefs, and behavioral intentions to adopt AI-based educational technologies. All constructs were operationalized as latent psychological variables reflecting teachers’ cognitive appraisals, affective responses, social perceptions, and motivational beliefs regarding AI adoption. The questionnaire comprised 13 latent constructs: digital leadership, subjective norm, experience, perceived enjoyment, anxiety, self-efficacy, perceived ease of use, perceived usefulness, attitude, facilitating conditions, price value, habit, and behavioral intention.

All constructs were adapted from validated scales grounded in the GETAMEL and UTAUT2 frameworks and prior AI adoption research (Abdullah and Ward, 2016; Venkatesh et al., 2012). Minor contextual adaptations were made to align items with AI-based educational technologies while preserving the original construct meaning. Digital leadership was measured using five school-context–specific items, while the remaining constructs were each measured with three items, yielding a total of 41 items.

The original English instruments were translated into Turkish and back-translated by bilingual experts to ensure linguistic and conceptual equivalence. The draft questionnaire was reviewed by academic specialists for clarity and content validity. Content validity was evaluated by three experts in educational psychology and educational technology, who confirmed the conceptual alignment of items with the proposed constructs. The instrument was subsequently pilot-tested with teachers to assess readability and reliability. Minor wording revisions were made, and pilot data were excluded from the final analysis.

All items were rated on a five-point Likert scale (1 = Strongly Disagree to 5 = Strongly Agree), with higher scores indicating stronger perceptions of digital leadership, more positive acceptance beliefs, and higher intention to adopt AI-based educational technologies. Details of the constructs, item loadings, and sources are reported in Table 1.

**Table 1:** Measurement items, factor loadings, and reliability/validity indicators.

Constructs and statements (adapted from study proposal)	FL	*α*	AVE	CR
Digital leadership (DL)		0.89	0.62	0.89
1. My school principal encourages the use of new technologies in lessons.	0.82			
2. My school principal motivates me to use AI-based applications.	0.85			
3. My school principal provides access to necessary AI-based resources (hardware/software).	0.77			
4. My school principal facilitates professional development opportunities for AI tools.	0.75			
5. My school principal articulates a clear vision for digital innovation in the school.	0.80			
Perceived usefulness (PU)		0.86	0.67	0.86
1. Using AI-based educational technologies helps me perform my job (teaching/assessment) more effectively.	0.84			
2. Using AI-based educational technologies increases my productivity.	0.81			
3. AI-based educational technologies are useful in enhancing student learning outcomes.	0.82			
Perceived ease of use (PEOU)		0.88	0.71	0.88
1. Learning to use AI-based educational technologies is easy for me.	0.86			
2. My interaction with AI-based educational technologies is clear and understandable.	0.83			
3. It would be easy for me to become skillful at using AI-based educational technologies.	0.88			
Self-efficacy (SE)		0.87	0.69	0.87
1. I feel confident in my ability to use AI tools for instructional purposes.	0.80			
2. I can use AI-based educational technologies even if there is no one nearby to help me.	0.86			
3. I have the necessary skills to effectively integrate AI into my teaching and assessment.	0.84			
Anxiety (AN)		0.85	0.66	0.85
1. I feel nervous when I think about using complex AI-based educational tools.	0.83			
2. I worry that I may make mistakes I cannot fix when using AI tools.	0.80			
3. Using AI-based educational technologies is somewhat intimidating to me.	0.82			
Subjective norm (SN)		0.83	0.62	0.83
1. My colleagues believe that I should use AI-based educational technologies.	0.78			
2. School administrators expect me to use AI-based educational technologies in my practice.	0.80			
3. The majority of my peers use AI-based educational technologies.	0.76			
Experience (EXP)		0.81	0.59	0.81
1. I have prior experience using similar digital tools in my teaching.	0.75			
2. I feel I am familiar with the concepts behind AI-based educational technologies.	0.78			
3. I have used AI-based tools for teaching or assessment at least once.	0.77			
Perceived enjoyment (PE)		0.84	0.64	0.84
1. I find the process of using AI-based educational technologies to be enjoyable.	0.81			
2. Using AI-based educational technologies is fun for me.	0.79			
3. I am interested in exploring the capabilities of AI-based educational technologies.	0.83			
Attitude (ATT)		0.89	0.73	0.89
1. I have a positive overall opinion about using AI-based educational technologies.	0.87			
2. Using AI-based educational technologies is a good idea.	0.85			
3. I like the idea of integrating AI into my instructional practice.	0.86			
Facilitating conditions (FC)		0.87	0.69	0.87
1. I have the technical infrastructure (e.g., internet, devices) needed to use AI tools.	0.83			
2. Specific help is available for me to use AI-based educational technologies when I have technical problems.	0.85			
3. AI-based educational technologies are compatible with the resources I already use.	0.82			
Price value (PV)		0.82	0.60	0.82
1. The benefits of using AI-based educational technologies outweigh the time/effort costs.	0.77			
2. Using AI-based educational technologies is cost-effective (financial, time, cognitive) for my school.	0.80			
3. AI-based educational technologies offer good value relative to the demands of my job.	0.75			
Habit (HAB)		0.85	0.66	0.85
1. I would feel strange if I did not use AI-based educational technologies in the future.	0.80			
2. Using AI-based educational technologies would become a natural part of my teaching routine.	0.84			
3. I have established routines for using digital tools similar to AI in my professional work.	0.82			
Behavioral intention (BI)		0.88	0.71	0.88
1. I intend to use AI-based educational technologies in my instruction and assessment processes.	0.85			
2. I predict I will use AI-based educational technologies frequently in the future.	0.87			
3. I plan to actively seek out and adopt AI-based educational technologies.	0.83			

### 3.3 Data analysis strategy and measurement validation

The proposed integrated model combining Digital Leadership, UTAUT2, and GETAMEL was tested using a two-stage Structural Equation Modeling (SEM) approach with SPSS and AMOS 28.0. First, the measurement model was assessed, followed by structural hypothesis testing. SEM was selected due to its suitability for examining complex relationships among latent constructs while accounting for measurement error. Maximum Likelihood estimation was employed, and this method was deemed appropriate as assumptions of normality were satisfied. Bootstrapping with 5,000 resamples was conducted to estimate indirect effects and generate bias-corrected 95% confidence intervals.

Confirmatory Factor Analysis (CFA) using Maximum Likelihood estimation was conducted to evaluate the measurement model comprising 41 items and 13 latent constructs. The model demonstrated good fit to the data (*χ*^2^/d*f* = 2.61; RMSEA = 0.051; CFI = 0.94; IFI = 0.93; TLI = 0.92), indicating that all items loaded appropriately on their intended constructs.

Given that all variables were measured using self-report instruments collected at a single time point, potential common method variance (CMV) was assessed. A single-factor CFA model was tested and demonstrated poor fit compared to the proposed measurement model, indicating that common method bias was not a substantial concern in the present study.

Reliability and validity were subsequently established. Internal consistency was satisfactory, with Cronbach’s alpha values ranging from 0.81 to 0.89 and Composite Reliability values exceeding the recommended threshold of 0.70. Convergent validity was confirmed as all Average Variance Extracted (AVE) values ranged from 0.59 to 0.73. Discriminant validity was supported, as the square root of each construct’s AVE exceeded its correlations with other constructs. Together, these results indicate that the measurement model was reliable and valid, providing a robust basis for testing the structural relationships.

To evaluate the comparative explanatory power of the proposed framework, nested model comparisons were conducted using fit indices and explained variance (*R*^2^) values. The integrated model was benchmarked against standalone UTAUT2 and GETAMEL models to assess incremental improvement in explanatory capacity. Descriptive statistics, inter-construct correlations, and validity indicators are presented in Table 2.

**Table 2:** Descriptive statistics, inter-construct correlations, and discriminant validity assessment.

Constructs	Mean	SD	DL	PU	PEOU	SE	ANX	SN	EXP	PE	ATT	FC	PV	HAB	BI
1. Digital leadership (DL)	4.15	0.85	**0.79**												
2. Perceived usefulness (PU)	4.35	0.79	0.58	**0.82**											
3. Perceived ease of use (PEOU)	4.22	0.81	0.49	0.66	**0.84**										
4. Self-efficacy (SE)	4.05	0.88	0.51	0.59	0.63	**0.83**									
5. Anxiety (ANX)	2.55	1.12	−0.45	−0.38	−0.42	−0.53	**0.81**								
6. Subjective norm (SN)	4.41	0.75	0.55	0.46	0.40	0.44	−0.21	**0.80**							
7. Experience (EXP)	3.88	0.95	0.40	0.45	0.48	0.50	−0.33	0.35	**0.77**						
8. Perceived enjoyment (PE)	4.18	0.83	0.48	0.55	0.51	0.46	−0.30	0.38	0.42	**0.81**					
9. Attitude (ATT)	4.45	0.77	0.52	0.68	0.60	0.61	−0.35	0.45	0.47	0.58	**0.85**				
10. Facilitating conditions (FC)	4.01	0.90	0.59	0.51	0.54	0.55	−0.41	0.39	0.46	0.42	0.50	**0.84**			
11. Price value (PV)	3.95	0.92	0.41	0.48	0.44	0.40	−0.25	0.30	0.38	0.35	0.44	0.40	**0.78**		
12. Habit (HAB)	3.70	0.99	0.35	0.42	0.39	0.43	−0.33	0.32	0.41	0.40	0.45	0.40	0.35	**0.81**	
13. Behavioral intention (BI)	4.55	0.70	0.56	0.72	0.68	0.65	−0.40	0.48	0.50	0.57	0.75	0.55	0.45	0.48	**0.86**

Bold diagonal values represent the square root of the Average Variance Extracted (AVE) for each construct. Off-diagonal values represent inter-construct correlations.

## 4 Findings

### 4.1 Structural model fit indices and comparative explanatory power

The proposed integrated Digital Leadership–UTAUT2–GETAMEL model was evaluated using SEM and demonstrated an excellent fit to the data. Model fit indices met or exceeded recommended thresholds (*χ*^2^/d*f* = 2.59; RMSEA = 0.048; SRMR = 0.045; CFI = 0.95; TLI = 0.94; IFI = 0.93), supporting the validity of the hypothesized structure.

To assess its comparative explanatory power, the integrated model was benchmarked against standalone UTAUT2 and GETAMEL models. The results show that the integrated framework explains 61% of the variance in teachers’ behavioral intention to adopt AI-based educational technologies (*R*^2^ = 0.61), substantially exceeding the explanatory power of the GETAMEL-only (*R*^2^ = 0.53) and UTAUT2-only (*R*^2^ = 0.45) models (see Table 3). These findings confirm the theoretical advantage of incorporating digital leadership alongside individual acceptance mechanisms.

**Table 3:** Comparison of model fit indices and explanatory power.

Model specification	*χ*^2^/d*f*	RMSEA	SRMR	CFI	TLI	IFI	*R* ^2^
Proposed integrated model	2.59	0.048	0.045	0.95	0.94	0.93	0.61
GETAMEL (original)	2.95	0.052	0.049	0.92	0.90	0.91	0.53
UTAUT2 (original)	3.15	0.055	0.053	0.90	0.88	0.89	0.45

### 4.2 Path analysis results for proposed models

The first phase of the analysis focused on the foundational role of Digital Leadership, addressing hypotheses H1–H6. The results substantiated that Digital Leadership is a critical organizational catalyst, exerting a significant positive influence on Subjective Norm (*β* = 0.42, *p* < 0.001), Experience (*β* = 0.38, *p* < 0.001), Perceived Enjoyment (*β* = 0.35, *p* < 0.001), and Self-Efficacy (*β* = 0.45, *p* < 0.001). Crucially, Digital Leadership had a significant negative impact on Anxiety (*β* = −0.31, *p* < 0.001), supporting H4, and a direct positive effect on Behavioral Intention (*β* = 0.22, *p* < 0.01), supporting H6. These findings suggest that school principals who actively support digital innovation not only directly encourage adoption but also significantly enhance teachers’ confidence, social motivation, and emotional readiness while reducing technology-induced apprehension.

The second phase examined the antecedents of Perceived Usefulness (PU) and Perceived Ease of Use (PEOU), addressing the internal GETAMEL mechanisms (H7–H16). The analysis confirmed that Subjective Norm (*β* = 0.33, *p* < 0.01), Experience (*β* = 0.28, *p* < 0.01), Perceived Enjoyment (β = 0.31, *p* < 0.001), and Self-Efficacy (*β* = 0.36, *p* < 0.001) significantly and positively predicted Perceived Usefulness. Conversely, Anxiety (*β* = −0.24, *p* < 0.01) had a significant negative effect on PU. A similar pattern was observed for Perceived Ease of Use, where Self-Efficacy (*β* = 0.41, *p* < 0.001) emerged as the strongest predictor, followed by Perceived Enjoyment (*β* = 0.39, *p* < 0.001), Experience (*β* = 0.30, *p* < 0.01), and Subjective Norm (*β* = 0.27, *p* < 0.01), while Anxiety exerted a strong negative influence (*β* = −0.33, *p* < 0.001). These results imply that teachers who feel supported by their peers, possess higher self-belief, and find AI interactions enjoyable are far more likely to perceive AI tools as both useful and easy to master.

The third phase evaluated the core acceptance pathways and contextual UTAUT2 determinants (H17–H24). As hypothesized, Perceived Ease of Use significantly impacted Perceived Usefulness (*β* = 0.48, *p* < 0.001) and Attitude (*β* = 0.44, *p* < 0.001). Furthermore, Perceived Usefulness strongly influenced Attitude (*β* = 0.52, *p* < 0.001) and Behavioral Intention (*β* = 0.41, *p* < 0.001), while Attitude was a robust predictor of Intention (*β* = 0.49, *p* < 0.001). Regarding the contextual factors, Facilitating Conditions (*β* = 0.29, *p* < 0.01), Price Value (*β* = 0.21, *p* < 0.05), and Habit (*β* = 0.34, *p* < 0.001) all demonstrated significant positive effects on Behavioral Intention. This indicates that beyond cognitive appraisal, the availability of resources, perceived value-for-effort, and established routinization are essential drivers of AI adoption.

**Figure 2:**
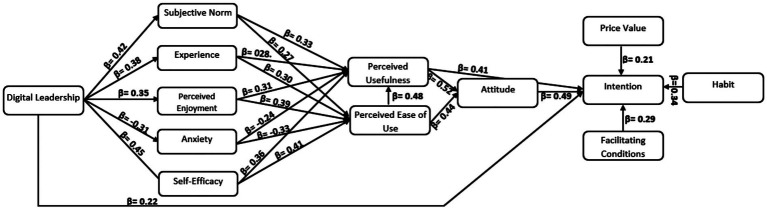
Integrated structural model of digital leadership, UTAUT2, and GETAMEL.

In terms of explanatory power, the proposed integrated model accounted for 61% of the total variance in teachers’ Behavioral Intention to adopt AI-based educational technologies (*R*^2^ = 0.61). Additionally, the model explained substantial variance in the mediating constructs: Attitude (*R*^2^ = 0.55), Perceived Usefulness (*R*^2^ = 0.49), and Perceived Ease of Use (*R*^2^ = 0.44) (see Table 4 and Figure 2).

**Table 4:** Path coefficients and hypothesis testing results.

Hypothesis	Path relationship	*β*	*t*-value	*p*	Result
Digital leadership effects					
H1	Digital leadership → subjective norm	0.42	6.85	***	Supported
H2	Digital leadership → experience	0.38	5.92	***	Supported
H3	Digital leadership → perceived enjoyment	0.35	5.41	***	Supported
H4	Digital leadership → anxiety	−0.31	4.88	***	Supported
H5	Digital leadership → self-efficacy	0.45	7.12	***	Supported
H6	Digital leadership → behavioral intention	0.22	3.15	**	Supported
Antecedents of PU and PEOU (GETAMEL)					
H7	Subjective norm → perceived usefulness	0.33	4.67	**	Supported
H8	Subjective norm → perceived ease of use	0.27	3.98	**	Supported
H9	Experience → perceived usefulness	0.28	4.10	**	Supported
H10	Experience → perceived ease of use	0.30	4.35	**	Supported
H11	Perceived enjoyment → perceived usefulness	0.31	4.52	***	Supported
H12	Perceived enjoyment → perceived ease of use	0.39	5.76	***	Supported
H13	Anxiety → perceived usefulness	−0.24	3.44	**	Supported
H14	Anxiety → perceived ease of use	−0.33	4.81	***	Supported
H15	Self-efficacy → perceived usefulness	0.36	5.23	***	Supported
H16	Self-efficacy → perceived ease of use	0.41	6.01	***	Supported
Core acceptance paths and UTAUT2 context					
H17	Perceived ease of use → perceived usefulness	0.48	7.45	***	Supported
H18	Perceived ease of use → attitude	0.44	6.90	***	Supported
H19	Perceived usefulness → attitude	0.52	8.12	***	Supported
H20	Perceived usefulness → behavioral intention	0.41	6.33	***	Supported
H21	Attitude → behavioral intention	0.49	7.65	***	Supported
H22	Price value → behavioral intention	0.21	2.58	*	Supported
H23	Habit → behavioral intention	0.34	4.77	***	Supported
H24	Facilitating conditions → behavioral intention	0.29	3.89	**	Supported
Explanatory power (variance explained)	Endogenous construct	*R* ^2^	Result		
	Behavioral intention	0.61	Substantial		
	Attitude	0.55	Moderate		
	Perceived usefulness	0.49	Moderate		
	Perceived ease of use	0.44	Moderate		

*p < 0.05; **p < 0.01; ***p < 0.001. All hypotheses were supported by the empirical data. *R*^2^ values indicate the proportion of variance in the dependent variable explained by the model.

## 5 Discussion

### 5.1 Summary of results

This study aimed to develop and validate an integrated framework linking Digital Leadership with UTAUT2 and GETAMEL to explain teachers’ adoption of AI-based educational technologies. The findings provide strong empirical support for the proposed model and demonstrate how environmental determinants interact with cognitive, affective, and contextual mechanisms to shape teachers’ behavioral intentions toward AI use. By integrating leadership as an environmental antecedent with established technology acceptance models, the study advances a multilevel psychological explanation of AI adoption in educational settings.

First, addressing RQ3, the integrated Digital Leadership–UTAUT2–GETAMEL model showed substantially higher predictive power than standalone models, explaining 61% of the variance in teachers’ behavioral intention to adopt AI. This finding suggests that AI adoption cannot be sufficiently understood through individual belief-based models alone but must incorporate environmental influences that shape belief formation and emotional regulation processes. The superior explanatory power of the integrated model supports the argument that cognitive appraisals (e.g., usefulness and ease of use), affective responses (e.g., enjoyment and anxiety), and perceived environmental support operate jointly in predicting intention.

Second, in response to RQ1, digital leadership emerged as a critical environmental antecedent of the cognitive–affective components of teachers’ readiness for AI adoption. Specifically, principals’ digital leadership significantly strengthened teachers’ mastery expectations (self-efficacy), enhanced intrinsic motivation (perceived enjoyment), reinforced perceived social pressure (subjective norm), and reduced threat appraisals (technology-related anxiety). Digital leadership also exerted a direct positive effect on behavioral intention, suggesting that leadership behaviors influence both mediated psychological pathways and more immediate motivational processes. These findings indicate that teachers’ readiness for AI adoption is best understood not as a standalone latent construct, but as a configuration of interrelated cognitive and affective mechanisms shaped by leadership and contextual support.

Third, addressing RQ2, the results clarified the cognitive–affective and contextual pathways underlying adoption intentions. Consistent with GETAMEL, teachers’ emotional and experiential states—particularly high self-efficacy, high enjoyment, and low anxiety—were central in shaping perceptions of usefulness and ease of use. These findings underscore the importance of cognitive appraisal processes and emotional regulation in determining technology acceptance. UTAUT2 further demonstrated that habit, facilitating conditions, and price value directly influenced intention, indicating that AI adoption depends not only on cognitive evaluations of utility but also on behavioral routinization, perceived environmental support, and broader cost–benefit appraisals. In this study, price value appears to reflect teachers’ evaluations of whether the pedagogical benefits of AI justify the required time, cognitive effort, workflow adjustment, and instructional demands, rather than direct personal monetary expenditure alone. Together, these mechanisms illustrate how intention formation in AI adoption reflects an interplay between environmental conditions, psychological processes, and emerging behavioral automaticity.

### 5.2 Theoretical implications

This study makes several important theoretical contributions by advancing a multilevel psychological explanation of AI adoption in educational settings. Rather than treating AI integration as a purely technological or administrative process, the findings demonstrate how environmental determinants, cognitive appraisals, affective responses, and behavioral consolidation mechanisms jointly shape teachers’ behavioral intentions. Importantly, the study does not treat psychological readiness as a separate latent variable. Instead, it conceptualizes readiness as an interpretive cognitive–affective configuration reflected in teachers’ self-efficacy, anxiety, perceived enjoyment, subjective norm, and related usefulness/ease-of-use appraisals. This clarification helps distinguish the present model from prior studies that invoke readiness in general terms without specifying how it is represented theoretically.

First, the study extends established technology acceptance frameworks by integrating Digital Leadership with UTAUT2 and GETAMEL within a unified psychological model. While prior research has largely focused on individual and technological determinants of AI and immersive technology adoption (e.g., Köroğlu, 2025; Konca et al., 2025), this study conceptualizes leadership as an environmental antecedent influencing belief formation and emotional regulation processes. The integrated model explains 61% of the variance in teachers’ behavioral intention—substantially exceeding the explanatory power typically reported for UTAUT2 or GETAMEL alone. More specifically, the novelty of the model lies in three features: the integration of Digital Leadership with UTAUT2 and GETAMEL in a single framework; the modeling of leadership as an antecedent of specific cognitive–affective mechanisms rather than as generic contextual support; and the interpretation of teachers’ psychological readiness as a configuration of self-efficacy, anxiety, perceived enjoyment, subjective norm, and related appraisal processes rather than as an unobserved standalone construct. This finding supports the argument that intention formation reflects the interaction of environmental influences with cognitive–affective mechanisms, responding to recent calls for more holistic, multilevel models of technology adoption (Zárate-Torres et al., 2025).

Second, the findings bridge macro-level environmental influences and micro-level psychological processes by grounding digital leadership effects in Social Cognitive Theory. Digital leadership significantly predicted self-efficacy and anxiety, indicating that leadership behaviors shape mastery expectations and threat appraisals during AI integration (Bandura, 1986; Okunlola and Naicker, 2025). This extends Digital Leadership Theory by demonstrating that leaders influence not only structural conditions but also teachers’ internal regulatory processes, thereby clarifying the psychological pathways through which environmental context affects behavioral intention.

Third, the study refines Self-Determination Theory in the context of AI adoption by highlighting the central roles of intrinsic motivation (perceived enjoyment) and anxiety regulation. Unlike traditional instructional technologies, AI systems often evoke heightened uncertainty and complexity-related concerns. The strong negative effect of anxiety alongside the positive effect of enjoyment underscores the importance of emotional regulation and intrinsic motivation in shaping cognitive appraisals of usefulness and ease of use. These findings challenge purely utility-based adoption models and align with emerging evidence that affective mechanisms are critical for sustained engagement with AI technologies (Frenkenberg and Hochman, 2025; Wang et al., 2025).

Finally, the study enriches UTAUT2 by offering a psychological reinterpretation of habit and price value in AI contexts. The significant role of habit suggests that adoption involves behavioral routinization and the development of automaticity, rather than episodic or trial-based use. Similarly, the effect of price value reflects cognitive cost–benefit appraisal processes in which teachers evaluate mental effort, time investment, and pedagogical payoff rather than solely financial cost. This interpretation is especially important in the context of public schools, where teachers typically do not directly pay for technologies provided by the institution. The continued significance of price value suggests that teachers evaluated AI adoption in terms of perceived net value rather than personal out-of-pocket cost. As reflected in the measurement items, this appraisal likely involved judgments about whether the pedagogical benefits of AI were sufficient to justify the additional time, cognitive load, workflow adaptation, and school-level resource implications associated with implementation. In early-stage AI adoption, such evaluations may also include indirect costs such as learning time, output verification, and lesson redesign. This nuanced interpretation extends UTAUT2 to complex, high-effort technologies and highlights the importance of cognitive and motivational mechanisms in sustaining AI integration.

### 5.3 Practical implications

The findings of this study offer evidence-informed implications for educational policymakers, school administrators, and teacher educators seeking to support the sustainable integration of AI in schools. These implications derive directly from the identified cognitive, affective, and contextual mechanisms underlying teachers’ adoption intentions. In practical terms, the results suggest that effective digital leadership should be translated into a school-level action package rather than remaining at the level of general encouragement. Consistent with the theoretical framework, these leadership actions can be operationalized through structured training, local support, low-risk experimentation, and incentive mechanisms.

#### 5.3.1 Tiered AI training

Because digital leadership significantly reduced anxiety and strengthened self-efficacy, school principals should organize tiered professional development aligned with teachers’ existing familiarity, confidence, and prior experience with AI. At the introductory level, training may focus on basic generative AI use, prompting strategies, and simple classroom use cases. At the intermediate level, training may address AI-supported lesson design, formative assessment, feedback generation, and instructional material adaptation. At the advanced level, principals should provide training on ethics, data privacy, bias, verification of AI-generated outputs, and the critical evaluation of pedagogical appropriateness. Such tiered structures may help teachers progress from initial exposure to more confident and pedagogically responsible AI use.

#### 5.3.2 School-based support structures

The findings also indicate that teachers’ adoption intentions are strengthened when support is available within the school environment. Accordingly, principals may designate an AI mentor teacher, instructional technology lead, or AI coordinator to provide ongoing guidance. This support can be operationalized through school-based office hours, peer coaching sessions, follow-up clinics, and collaborative planning meetings. Providing protected time for teacher collaboration is especially important, as it enables teachers to ask questions, troubleshoot challenges, and exchange practical experiences in a low-pressure setting. Such local support structures may reduce uncertainty while strengthening both facilitating conditions and perceived competence.

#### 5.3.3 Low-risk experimentation opportunities

Because anxiety and perceived complexity remain important barriers, school leaders should normalize low-risk experimentation before expecting school-wide implementation. One practical strategy is to begin with small pilot teams or volunteer teacher groups that test selected AI tools in limited instructional contexts. Principals may also provide curated lists of approved AI tools and create sandbox environments in which teachers can explore applications without immediate performance pressure. This staged approach allows schools to identify workable practices, generate local examples of successful implementation, and reduce threat appraisals associated with unfamiliar technologies. In this way, experimentation becomes structured, supported, and pedagogically purposeful rather than informal or risky.

#### 5.3.4 Incentives and recognition mechanisms

The significance of price value suggests that teachers evaluate AI adoption in relation to effort, time, and professional payoff. For this reason, principals should support AI integration not only through training but also through incentives that make participation worthwhile. These may include release time for experimentation and collaborative planning, certificates or micro-credentials for completed training, visible recognition of innovative classroom practices, and small internal innovation grants for pilot projects. Schools may also require or encourage participating teachers to share effective practices through internal workshops, demonstration lessons, or peer-learning sessions. Such incentive mechanisms may increase perceived value, reinforce sustained engagement, and help AI use become integrated into routine professional practice.

Taken together, these findings suggest that school principals can build effective digital leadership through a coherent four-part strategy: differentiated training, school-based support, low-risk experimentation, and meaningful incentives. These actions provide the practical counterpart to the leadership processes identified in the theoretical model, translating digital vision, professional learning, resource allocation, experimentation, and supportive expectations into implementable school practices. This type of structured action package is likely to strengthen teachers’ self-efficacy, reduce anxiety, improve perceived value, and support the sustained pedagogical integration of AI in schools.

### 5.4 Limitations and recommendations for future research

While this study provides robust evidence for the proposed integrated model, several limitations should be acknowledged to guide future research.

First, the research employed a cross-sectional design, capturing teachers’ perceptions at a single point in time. While structural equation modeling allows for the testing of causal paths, it cannot definitively establish causality. Future studies should adopt longitudinal designs to track how digital leadership influences changes in teachers’ anxiety and adoption habits over an extended period, particularly as AI tools evolve.

Second, the data were collected exclusively from self-reported measures. While this is standard for assessing psychological constructs like “intention” and “attitude,” self-reports can be subject to social desirability bias. Future research could benefit from a mixed-methods approach, combining survey data with objective usage logs from learning management systems or classroom observations to verify actual adoption behaviors.

Third, the study employed a non-probability convenience sampling strategy accessed through school principals. Although this approach was appropriate for testing a theory-driven model and enabled the recruitment of a sufficiently large and heterogeneous sample across school levels and institutional contexts, it does not ensure statistical representativeness. Teachers and schools that were more open to digital innovation may have been more likely to participate, which may limit the extent to which the findings can be generalized to the broader teacher population in Türkiye. Accordingly, the findings should be interpreted as analytically generalizable to the proposed theoretical relationships rather than statistically generalizable to all teachers.

Fourth, the study treated “AI-based educational technologies” as a broad category. However, teachers’ reactions may vary significantly depending on the specific type of AI (e.g., Generative AI like ChatGPT vs. Diagnostic AI for assessment). Future research should investigate whether the drivers of adoption differ across these specific technology types.

Finally, the study was conducted within the specific cultural and educational context of Türkiye. Cultural factors such as power distance or uncertainty avoidance may influence the impact of leadership and social norms. Comparative studies involving other countries would be valuable to test the cross-cultural generalizability of the integrated Digital Leadership–UTAUT2–GETAMEL model.

## Data Availability

The original contributions presented in the study are included in the article/supplementary material, further inquiries can be directed to the corresponding author.

## References

[ref1] Abdullah F. Ward R. (2016). Developing a general extended technology acceptance model for E-learning (GETAMEL) by analysing commonly used external factors. Comput. Hum. Behav. 56, 238–256. doi: 10.1016/j.chb.2015.11.036

[ref2] Acosta-Enriquez B. G. Ballesteros M. A. A. de los Angeles Guzman Valle M. Angaspilco J. E. M. Blanco-García L. E. Ventura G. C. . (2025). Determinants of AI use in university teachers: the role of leadership, teaching concerns, and constructivist pedagogical beliefs. Human Behav. Emerg. Technol. 2025:4834893. doi: 10.1155/hbe2/4834893

[ref3] Aldraiweesh A. A. Alturki U. (2025). The influence of social support theory on AI acceptance: examining educational support and perceived usefulness using SEM analysis. IEEE Access 13, 18366–18385. doi: 10.1109/ACCESS.2025.3534099

[ref4] Alkan B. B. Inal G. Karakus L. Alkan N. (2025). Shaping the future of education: school principals’ views on AI, big data and robot teachers. AI & Soc 41, 1417–1433. doi: 10.1007/s00146-025-02570-w

[ref5] An X. Chai C. S. Li Y. Zhou Y. Shen X. Zheng C. . (2023). Modeling English teachers’ behavioral intention to use artificial intelligence in middle schools. Educ. Inf. Technol. 28, 5187–5208. doi: 10.1007/s10639-022-11286-z

[ref6] Ateş H. (2025). Integrating augmented reality into intelligent tutoring systems to enhance science education outcomes. Educ. Inf. Technol. 30, 4435–4470. doi: 10.1007/s10639-024-12970-y

[ref7] Ateş H. Gündüzalp C. (2025). Proposing a conceptual model for the adoption of artificial intelligence by teachers in STEM education. Interact. Learn. Environ. 33, 4020–4046. doi: 10.1080/10494820.2025.2457350, 37339054

[ref8] Bandura A. (1986). Social Foundations of Thought and Action: A Social Cognitive Theory. Hoboken, NJ: Prentice-Hall.

[ref9] Basarmak U. Ates H. (2026). Integrating UTAUT-2 and protection motivation theory to explain pre-service teachers’ adoption of AI-supported game-based learning. Educ. Inf. Technol. doi: 10.1007/s10639-026-13975-5

[ref10] Berkovich I. Hassan T. (2024). Principals’ digital instructional leadership during the pandemic: impact on teachers’ intrinsic motivation and students’ learning. Education. Manag. Admin. Leader. 52, 934–954. doi: 10.1177/17411432221113411

[ref11] Biswas M. Murray J. (2024). The impact of education level on AI reliance, habit formation, and usage. In Proceedings of the 2024 29th International Conference on Automation and Computing (ICAC) (pp. 1–6). New York, NY: IEEE

[ref12] Bixler K. Ceballos M. (2025). Principals leading AI in schools for instructional leadership: a conceptual model for principal AI use. Leadersh. Policy Sch. 24, 137–154. doi: 10.1080/15700763.2024.2428297

[ref13] Celik I. Muukkonen H. Siklander S. (2025). Teacher–artificial intelligence (AI) interaction: the role of trust, subjective norm and innovativeness in teachers’ acceptance of educational chatbots. Policy Futures Educ. 24:14782103251348551. doi: 10.1177/14782103251348551

[ref14] Chen J. J. Delaney V. (2025). Leveraging AI to enhance children’s learning: anchoring policy and practice in equity, AI literacy, and ethics for education leaders and teachers. Early Childhood Educ. J. 54, 2655–2665. doi: 10.1007/s10643-025-02036-0

[ref15] Choi S. Jeon J. Jang Y. (2025). Exploring teacher intention to teach AI: self-determination theory (SDT) and motivation-opportunity-ability (MOA) perspectives. Educ. Inf. Technol. 30, 24173–24200. doi: 10.1007/s10639-025-13693-4, 30311153

[ref16] Chou C. M. Shen T. C. Shen T. C. Shen C. H. (2024). Teachers’ adoption of AI-supported teaching behavior and its influencing factors: using structural equation modeling. J. Comput. Educ. 12, 853–896. doi: 10.1007/s40692-024-00332-z

[ref17] Dasruth J. Naicker S. R. Smith C. (2024). Teachers’ perceptions of principals’ digital leadership practices in a school district in a developing country. Soc. Sci. Humanit. Open 10:101192. doi: 10.1016/j.ssaho.2024.101192

[ref18] David L. Weinstein N. (2024). Using technology to make learning fun: technology use is best made fun and challenging to optimize intrinsic motivation and engagement. Eur. J. Psychol. Educ. 39, 1441–1463. doi: 10.1007/s10212-023-00734-0

[ref19] Dringó-Horváth I. Rajki Z. Nagy J. (2025). University teachers’ digital competence and AI literacy: moderating role of gender, age, experience, and discipline. Educ. Sci. 15:868. doi: 10.3390/educsci15070868

[ref20] Du Y. Gao H. (2022). Determinants affecting teachers’ adoption of AI-based applications in EFL context: an analysis of analytic hierarchy process. Educ. Inf. Technol. 27, 9357–9384. doi: 10.1007/s10639-022-11001-y

[ref21] Elyakim N. (2025). Bridging expectations and reality: addressing the price-value paradox in teachers’ AI integration. Educ. Inf. Technol. 30, 16929–16968. doi: 10.1007/s10639-025-13466-z, 30311153

[ref22] Er-Rafyg A. Zankadi H. Idrissi A. (2024). “AI in adaptive learning: challenges and opportunities,” in Modern Artificial Intelligence and Data Science 2024: Tools, Techniques and Systems, ed. Idrissi A. (Berlin: Springer), 329–342.

[ref23] Farmanesh P. Vehbi A. Solati Dehkordi N. (2025). Uprooting technostress: digital leadership empowering employee well-being in the era of industry 4.0. Sustainability 17:8868. doi: 10.3390/su17198868

[ref24] Frenkenberg A. Hochman G. (2025). It’s scary to use it, it’s scary to refuse it: the psychological dimensions of AI adoption—anxiety, motives, and dependency. Systems 13:82. doi: 10.3390/systems13020082

[ref25] Fullan M. Azorín C. Harris A. Jones M. (2024). Artificial intelligence and school leadership: challenges, opportunities and implications. Sch. Leadersh. Manag. 44, 339–346. doi: 10.1080/13632434.2023.2246856

[ref26] Gambo I. Abegunde F. J. Gambo O. Ogundokun R. O. Babatunde A. N. Lee C. C. (2025). GRAD-AI: An automated grading tool for code assessment and feedback in programming course. Educ. Inf. Technol. 30, 9859–9899. doi: 10.1007/s10639-024-13218-5

[ref27] Gamlem S. M. McGrane J. Brandmo C. Moltudal S. Sun S. Z. Hopfenbeck T. N. (2025). Exploring pre-service teachers’ attitudes and experiences with generative AI: a mixed methods study in Norwegian teacher education. Educ. Psychol. 46, 27–51. doi: 10.1080/01443410.2025.2528663

[ref28] Gkanatsiou M. A. Triantari S. Tzartzas G. Gkanatsios S. Fragulis G. F. (2025). AI and digital tools: transforming mediation and leadership in higher education (HEIs). Eng. Proc. 107:104. doi: 10.3390/engproc2025107104

[ref29] Harsanti H. R. Sudibjo N. Riady S. Yu P. (2025). Exploring Indonesian teachers’ intention to use artificial intelligence in schools: the impact of digital leadership, digital readiness, and perceived usefulness. Cogent Soc. Sci. 11:2593596. doi: 10.1080/23311886.2025.2593596

[ref30] Hartley K. Hayak M. Ko U. H. (2024). Artificial intelligence supporting independent student learning: an evaluative case study of ChatGPT and learning to code. Educ. Sci. 14:120. doi: 10.3390/educsci14020120

[ref31] Herzallah A. M. Makaldy R. (2025). Technological self-efficacy and sense of coherence: key drivers in teachers’ AI acceptance and adoption. Comput. Educ. Artif. Intell. 8:100377. doi: 10.1016/j.caeai.2025.100377

[ref32] Hossain S. Fernando M. Akter S. (2025). Digital leadership: towards a dynamic managerial capability perspective of artificial intelligence-driven leader capabilities. J. Leadersh. Organ. Stud. 32, 189–208. doi: 10.1177/15480518251319624

[ref33] Hu L. Wang H. Xin Y. (2025). Factors influencing Chinese pre-service teachers’ adoption of generative AI in teaching: an empirical study based on UTAUT2 and PLS-SEM. Educ. Inf. Technol. 30, 12609–12631. doi: 10.1007/s10639-025-13353-7, 30311153

[ref34] Işik M. (2025). Impact of artificial intelligence and technology leadership on professional teacher development. J. Educ. Res. 118, 622–631. doi: 10.1080/00220671.2025.2510386, 37339054

[ref35] Jamarani A. Haddadi S. Sarvizadeh R. Haghi Kashani M. Akbari M. Moradi S. (2024). Big data and predictive analytics: a systematic review of applications. Artif. Intell. Rev. 57:176. doi: 10.1007/s10462-024-10811-5

[ref36] Jing M. Guo Z. Wu X. Yang Z. Wang X. (2025). Higher education digital academic leadership: perceptions and practices from Chinese university leaders. Educ. Sci. 15:606. doi: 10.3390/educsci15050606

[ref37] Karakose T. Polat H. Tülübaş T. Demirkol M. (2024). A review of the conceptual structure and evolution of digital leadership research in education. Educ. Sci. 14:1166. doi: 10.3390/educsci14111166

[ref38] Kim J. J. Soh J. Kadkol S. Solomon I. Yeh H. Srivatsa A. V. . (2025). AI anxiety: a comprehensive analysis of psychological factors and interventions. AI. Ethics 5, 3993–4009. doi: 10.1007/s43681-025-00686-9, 30311153

[ref39] Konca A. S. Simsar A. Alhajji R. Al Mansoori A. (2025). Cross-cultural perspectives on AI adoption in teacher education: a comparative study of pre-service teachers in Turkey and the United Arab Emirates. Interact. Learn. Environ. 33, 5820–5846. doi: 10.1080/10494820.2025.2488143, 37339054

[ref40] Köroğlu M. (2025). Pioneering virtual assessments: augmented reality and virtual reality adoption among teachers. Educ. Inf. Technol. 30, 9901–9948. doi: 10.1007/s10639-024-13159-z

[ref41] Kovacevic M. Dagen T. Rajter M. (2025). Leading AI-driven student engagement: the role of digital leadership in higher education. Educ. Sci. 15:775. doi: 10.3390/educsci15060775

[ref42] Li F. Ruijs N. Lu Y. (2022). Ethics & AI: a systematic review on ethical concerns and related strategies for designing with AI in healthcare. AI 4, 28–53. doi: 10.3390/ai4010003

[ref43] Lin Q. (2024). Digital leadership: a systematic literature review and future research agenda. Eur. J. Innov. Manag. 28, 2469–2488. doi: 10.1108/ejim-07-2023-0522

[ref44] Liu Y. Awang H. Mansor N. S. (2025). Exploring the potential barrier factors of AI Chatbot usage among teacher trainees: from the perspective of innovation resistance theory. Sustainability 17:4081. doi: 10.3390/su17094081

[ref45] Liu J. Dai Q. Chen J. (2025). Factors affecting teachers’ use of digital resources for teaching mathematical cultures: an extended UTAUT-2 model. Educ. Inf. Technol. 30, 7659–7688. doi: 10.1007/s10639-024-13105-z

[ref46] Okunlola J. O. Naicker S. R. (2025). Principals’ digital leadership competencies in the fourth industrial revolution: teachers’ perspectives. Educ. Sci. 15:656. doi: 10.3390/educsci15060656

[ref47] Omar M. N. Ismail S. N. (2021). Empowering teacher self-efficacy on ICT: how does technology leadership play a role? MOJEM Malays. Online J. Educ. Manag. 9, 1–22.

[ref48] Özdemir A. M. Ateş H. (2025). Harnessing holographic technology in science education: an integrated GETAMEL-TOE model analysis. Interact. Learn. Environ. 1–29. doi: 10.1080/10494820.2025.2564738

[ref49] Perez-Liebana D. Liu J. Khalifa A. Gaina R. D. Togelius J. Lucas S. M. (2019). General video game AI: a multitrack framework for evaluating agents, games, and content generation algorithms. IEEE Trans. Games 11, 195–214. doi: 10.1109/tg.2019.2901021

[ref50] Sagbas M. Oktaysoy O. Topcuoglu E. Kaygin E. Erdogan F. A. (2023). The mediating role of innovative behavior on the effect of digital leadership on intrapreneurship intention and job performance. Behavioral Sciences 13:874. doi: 10.3390/bs13100874, 37887524 PMC10604224

[ref51] Schmitz M. L. Antonietti C. Consoli T. Cattaneo A. Gonon P. Petko D. (2023). Transformational leadership for technology integration in schools: empowering teachers to use technology in a more demanding way. Comput. Educ. 204:104880. doi: 10.1016/j.compedu.2023.104880

[ref52] Suryanarayana K. S. Kandi V. P. Pavani G. Rao A. S. Rout S. Krishna T. S. R. (2024). Artificial intelligence enhanced digital learning for the sustainability of education management system. J. High Technol. Manag. Res. 35:100495. doi: 10.1016/j.hitech.2024.100495

[ref53] van Dun D. H. Kumar M. (2023). Social enablers of industry 4.0 technology adoption: transformational leadership and emotional intelligence. Int. J. Oper. Prod. Manag. 43, 152–182. doi: 10.1108/ijopm-06-2022-0370

[ref54] Venkatesh V. Thong J. Y. Xu X. (2012). Consumer acceptance and use of information technology: extending the unified theory of acceptance and use of technology. MIS Q. 36, 157–178. doi: 10.2307/41410412

[ref55] Vermeulen M. Van Acker F. Kreijns K. Van Buuren H. (2015). Does transformational leadership encourage teachers’ use of digital learning materials. Educ. Manag. Adm. Leadersh. 43, 1006–1025. doi: 10.1177/1741143214535749

[ref56] Wang Y. Zhao Y. Tian X. Yang J. Luo S. (2025). The influence of subjective knowledge, technophobia and perceived enjoyment on design students’ intention to use artificial intelligence design tools. Int. J. Technol. Des. Educ. 35, 333–358. doi: 10.1007/s10798-024-09897-3

[ref57] Xu X. (2025). “Technology and innovation: transforming educational leadership,” in Cultivating Inclusive Educational Leadership Ecosystems: Women Trailblazers and the Path Forward, eds. Yousefi M. Sorayyaei Azar A. Anderson C. Gray S. (Palmdale, PA: IGI Global Scientific Publishing), 33–86.

[ref58] Xue L. Ghazali N. Mahat J. (2025). A systematic review of UTAUT and UTAUT2 for AI adoption in education. Int. J. Hum. Comput. Interact. 42, 5666–5690. doi: 10.1080/10447318.2025.2552867

[ref59] Zaim M. Arsyad S. Waluyo B. Ardi H. Al Hafizh M. Zakiyah M. . (2024). AI-powered EFL pedagogy: integrating generative AI into university teaching preparation through UTAUT and activity theory. Comput. Educat. Artific. Intell. 7:100335. doi: 10.1016/j.caeai.2024.100335

[ref60] Zárate-Torres R. Rey-Sarmiento C. F. Acosta-Prado J. C. Gómez-Cruz N. A. Rodríguez Castro D. Y. Camargo J. (2025). Influence of leadership on human–artificial intelligence collaboration. Behavior. Sci. 15:873. doi: 10.3390/bs15070873, 40723657 PMC12292626

[ref61] Zhang P. Yang H. (2025). The GETAMEL model: features of the adaptation of teachers in the transition to on-line learning. Internat. J. Human–Computer Interact. 41, 102–114. doi: 10.1080/10447318.2023.2295694

